# A Chelating Resin of EDTA-Type Modified Cross-Linking Polystyrene to Preconcentrate Trace Metals for Seawater Detection

**DOI:** 10.1155/2022/2080600

**Published:** 2022-10-14

**Authors:** Chao Li, Linna Duan, Mingchao Han, Hongwei He, Mengxiang Yuan, Hairong Wang

**Affiliations:** ^1^Beihai Offshore Engineering Survey Institute, North China Sea Bureau of Ministry of Natural Resources, Qingdao 266033, China; ^2^College of Textiles & Clothing, Qingdao University, Qingdao 266071, Shandong, China; ^3^College of Environment and Civil Engineering, Chengdu University of Technology, Chengdu 610059, China

## Abstract

The determination of trace metals in seawater is an important project of marine environmental monitoring. However, the presence of many alkali metal ions with high concentration, such as sodium ion, seriously interferes with the detection limit and accuracy of atomic absorption spectrometry (AAS, flame/graphite furnace integrated). The conventional chemical methods for the enrichment of trace metals are complex, and low boiling point organic solvents are used. In this paper, a kind of commercial cross-linked polystyrene resin microspheres was chloromethylated and aminated to introduce EDTA-type amino polycarboxylic groups and then loaded in a column as the absorption filler. A set of seawater pretreatment and enrichment devices was designed and assembled. The enriching process and conditions of trace Cu, Zn, Pb, and Cd in standard seawater were studied. 10 g of the modified resin could enrich the equivalent seawater and remove successfully the light metal ions. pH = 5∼9 and 0.2 mL/min of the flow rate were the suitable conditions for preconcentration. The enriched metal ions in the eluent were analyzed on the AAS. Compared with the conventional solvent method, the novel material and enrichment device have high preconcentration efficiency, strong anti-interference ability, and low cost and could be directly applied for routine seawater detection.

## 1. Introduction

Trace amounts of trace metals are widely present in various waters, and common analytical methods are hard to detect them [1–5]. In particular, the measurement of trace trace metal ions is often subject to a large degree of interference because there is the presence of a higher concentration of alkali (Earth) metal ions in these waters, such as seawater, salt-lake water, and salty industrial wastewaters, which strongly affect the test results of atomic absorption spectrometry (AAS, flame/graphite furnace) being applied widely and accepted as a standard means. In fact, the salty sample to be sure of contents of trace trace metals must be pretreated to concentrate trace metal ions and remove alkali (Earth) metal ones before being tested on AAS. [6–8].

At present, the main methods for testing trace trace metal in seawater include some national standard methods (Chinese GB 17378.4–2007), mainly using ammonium pyrrolidine dithiocarbamate (APDC) and sodium diethyldithiocarbamate (DDTC) as complexing agents and organic solvent methyl isobutyl ketone (MIBK) as extraction media to concentrate trace metal ions [9–11]. However, this method has got a complicated operation process and uses much higher purity reagents, which is easy to introduce impurities and not economical. At the same time, the organic solvent is inevitably volatilized during the operation, which takes the deviation of the test concentration and does certain harms to operators and the environment as well. Therefore, in recent years, the concentration and enrichment of trace metals in high salt water, especially in seawater, have gradually been given more and more attention [12–14]. The core part (material) of a typical preconcentration system is a chelating column filled with functional resins. Minami et al. [15] and Biller and Bruland [16] developed a concentration system using Chelate-PA1 chelating resin, respectively, and Jiménez et al. [17] used Chelex 100 resin to build a similar concentration enrichment device for measuring trace metal ion content in seawater. However, the structures of commercial adsorption resins are not uncovered, and the performance robustness for the long-term use of the adsorption/desorption process of trace metal ions is not elucidated clearly. Ethylene diamine tetraacetic acid (EDTA) was a good chelating agent in chemistry, and EDTA-like or EDTA-modified recyclable resins had been designed and applied in removal of trace metal ions for purification of water [18, 19]. The stability of the obtained resins through polymerization by using acrylate monomers is insufficient for long-term use because there will be decomposition of the esters of acrylate to a certain extent when purged by acid or buffer solution during desorption, which may lead to the disintegration of the resin system. There were other chelating polymeric resins developed by introducing inorganic graphene [20], multiwalls carbon nanotubes (MWCNTs) [21], magnetic hollow porous oval shape NiFe_2_O_4_ [22] or organic ionic liquid [23], chitosan etc. [24]. Due to the fixedness and stability, these modified resins had no good recyclability and repeatability in the process of adsorption-desorption. Sometimes, there was only one or a few ions exhibiting a response of adsorption-desorption [24].

Polystyrene (PS) and divinyl benzene cross-linking resin is a sort of stable alkyl one having good resistance of acid, alkali, and organic solvents. Consequently, PS cross-linking resin is often modified to be a cationic or anionic adsorbent. In this work, the commercially and facilely obtained PS cross-linking resin beads were surface-modified and grafted with EDTA-type structure having polycarboxy and amino groups and used to preconcentrate trace metal salts from standard seawater samples. The self-built enrichment device was employed, and trace metal ions enriched successfully to remove the light metal ions, such as Na^+^ with high concentration, in which the trace Cu, Zn, Pb, and Cd were determined by a AAS integrated flame/graphite furnace, and the testing results coincided with the values given by the conventional organic extraction method.

## 2. Experimental

### 2.1. Reagents and Materials

The cross-linking PS beads were purchased from Dae (Tianjin) Technology Co., Ltd. Chloromethyl methyl ether (CMME), tin tetrachloride (SnCl_4_), ethylenediamine (EDA), 4-dimethylaminopyridine (DMAP), sodium chloroacetate, N,N-dimethyl formamide (DMF), trichloromethane (CHCl_3_), isobutyl ketone (MIBK), cyclohexane, and ethanol were of analytical grade, bought from Aladdin Co. (China), which were used without further purification. Nitric acid (HNO_3_), acetic acid (AcOH), and ammonium acetate (NH_4_Ac) were of trace metal grade, and deionized (ultrapure) water was given with a Milli-Q Integral 5 system.

Ammonium pyrrolidine dithiocarbamate (APDC) and sodium diethyldithiocarbamate (DDTC) as complexing agents (GB 17378.4–2007) with 99.9% purity were purchased from Aladdin Co. Ltd and used without further purification. Standard seawater was obtained from Beijing Putian Tongchuang BioTech Co., Ltd, and it contained mainly metal ions of Na (10.76 g/L), Mg (1.3 g/L), Ca (0.41 g/L), and K (0.40 g/L), and trace metals of Cu (5.0 ± 0.4 *µ*g/L), Zn (70 ± 3 *µ*g/L), Pb (10.0 ± 0.6 *µ*g/L), and Cd (1.00 ± 0.06 *µ*g/L).

### 2.2. Preparation of EDTA-Type Modified PS Beads

#### 2.2.1. Chloromethylation of PS Bead Surface (CPS), [25]

20 mL of CH_2_Cl_2_ and 3 g of PS beads were added in a 100 mL 4-neck round flask equipped with a condenser and magnetic stirrer at room temperature. After the solution was left under these conditions for 20 minutes, 7 mL of CMME was added dropwise with continuous stirring for 45 minutes. Then, 3 mL of CMME and 0.4 mL of anhydrous SnCl_4_ were added dropwise in 10 min to the mixture. The reaction was kept for a period of 8 hours. Then, the reaction was terminated, and the solid chloromethylated PS beads were afforded after filtration and washed three times by 50 mL × 3 of CHCl_3_.

#### 2.2.2. Amination of PS Bead Surface (APS)

20 mL of CH_2_Cl_2_, 2.4 g of DMAP, and 1.2 g of EDA were added in a 100 mL 4-neck round flask equipped with a magnetic stirrer at room temperature. After the solution was stirred for 20 minutes, 2.0 g of CPS beads were added with continuous stirring. The reaction was kept for a period of 8 hours. Then, the reaction was terminated, and the solid aminated PS beads were given after filtration and washed three times by 50 mL × 3 of CH_2_Cl_2_.

#### 2.2.3. Polycarboxyl Introduction to APS (CAPS)

**Scheme 1:**
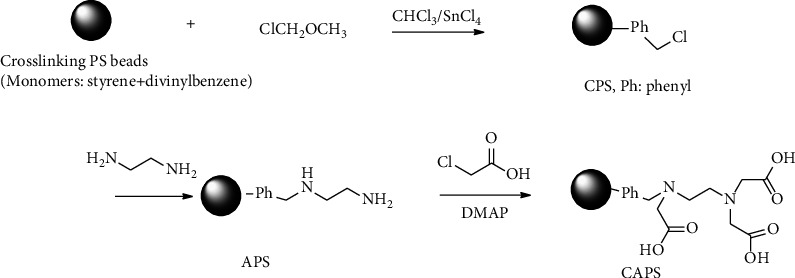
EDTA-type modified PS beads.

20 mL of DMF, 2.4 g of DMAP, and 2.3 g of sodium chloroacetate were added in a 100 mL 4-neck round flask equipped with a magnetic stirrer at room temperature. After the solution was stirred for 20 minutes, 2.0 g of APS beads were added with continuous stirring. The reaction was kept for a period of 24 hours. Then, the reaction was terminated, and the solid amino-polycarboxylic PS beads were given after filtration and washed three times by 50 mL × 3 of ethanol Scheme 1.

The given CAPS beads were observed by using a digital camera and scan electron microscope (SEM, TESCAN-VEGA3, USA). The structures of PS and CAPS beads were characterized by Fourier transform infrared (FT-IR, Nicolet iS10, Thermo Fisher Scientific, USA).

### 2.3. Enriching System and Concentration Process

**Figure 1:**
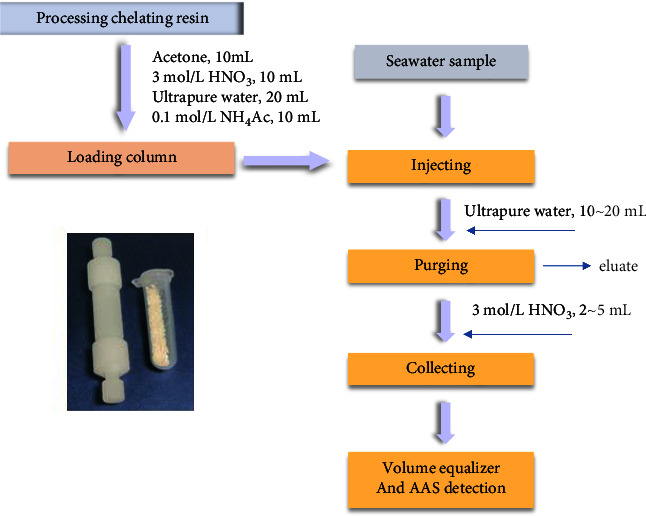
The process of trace metal ion preconcentration. Insert: the preconcentration column and filling resin, as-prepared EDTA-type CAPS.

Before loaded in a column, the amino polycarboxylic PS beads (CAPS chelating resin) were processed or washed by using acetone, 3 mol/L HNO_3_, ultrapure water, and 0.1 mol/L NH_4_Ac, shown in Figure 1. Then, over 8.0 g of the CAPS beads were loaded in a cylinder with 8 mm in inner diameter and 50 mm in length, and then the enriching system was built. The effect on enriching efficiency was explored, such as mass of resin, pH of seawater, and the flow rate.

A typical process was followed. 20 mL of the standard seawater sample was injected by using a pump (speed: 0.2 mL/min) and passed by using a 4-way valve and column filled with processed 10 g of CAPS resin, and then 10 mL of ultrapure water was injected to purge. The eluate during this period flowed down into a tank of “waste liquids.” Switching the 3-way valve to the mode of “eluate (HNO_3_)” and 4-way valve to no.3 inlet, the desorption process of trace metal ions by 2 mL of 1 mol/L HNO_3_ started, which also meant the regeneration of column. After purging of HNO_3_ liquid was accomplished, the HNO_3_ eluate was collected for determination of Cu, Zn, Pb, and Cd by means of AAS (PerkinElmer PinAAcle 900T, flame/graphite furnace integrated design with longitudinal Zeeman background subtraction system) and flame AAS for Zn and graphite furnace one for Cu, Pb, and Cr, respectively. The flame AAS has an air-acetylene burner used for determination of Zn. Spectral bandwidth (0.7 nm), the flow rate of acetylene gas (1.4 L/min), and the nebulizer flow rate (10.0 mL/min) are the conventional working parameters. For graphite furnace AAS, these parameters are spectral bandwidth (0.7 nm), lamp current 7 mA, ashing temperature 400°C, and atomization temperature 2300°C. Finally, the column was purged by 20 mL of deion water, 20 mL of buffer solution (0.05 M AcOH/AcONH_4_, pH 6), and 20 mL of deion water in turn.

To explore the effect of resin mass, the flow rate and pH of water sample on the enriching results of trace metals as well as the different enrichment conditions are shown in Table. 1. The samples to be enriched and tested were the standard seawater containing Zn, Cu, Pb, and Cd.

### 2.4. Determination of the Limit of Detection (LOD)

The standard seawater and its diluted samples (1/2, 1/4, 1/8, 1/10 of the original standard seawater sample in concentration) were processed and tested. 100 mL of every (diluted) seawater sample was enriched by 10 mL of HNO_3_ eluent for detection of AAS. The LOD was calculated from the curves of absorbances with concentration. Every sample was measured 10 times to determine the standard deviation of the blank. The absorbance of each enriching eluent was measured three times. The relationship of the average value of the absorbance intensity of every metal ion with the concentration of their eluent was built and plotted. The LOD was calculated using

(1) $$\begin{array}{c} LOD=\frac{3S_{d}}{k}, \end{array}$$

where *S*_d_ is the standard deviation of the blank experiments, and *k* is the slope value of the linearity plot.

## 3. Results and Discussion

### 3.1. Characterization of Polycarboxyl Amino Modified PS Beads

**Figure 2:**
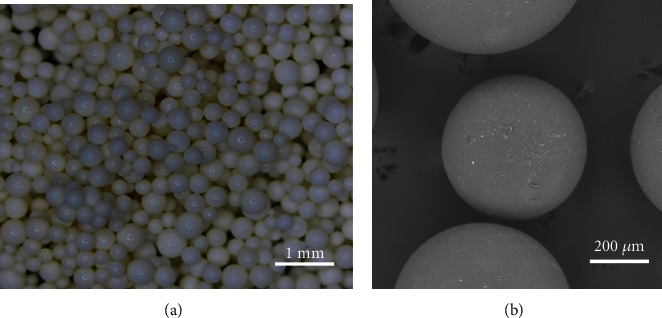
(a) Digital photo of CAPS beads and (b) its SEM picture.

**Figure 3:**
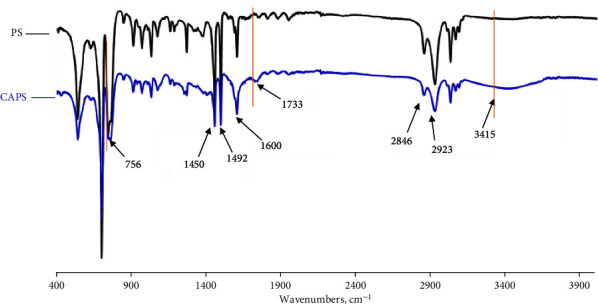
FT-IR spectra of cross-linking PS beads and polycarboxyl amino PS beads (CAPS).

As shown in Figure 2, the as-obtained pure PS beads and then EDTA-type modified ones, CAPS are regular balls and have a smooth surface, whose average size is 400–600 *μ*m in diameter. The structures of these beads were characterized by means of FT-IR (KBr pellets), as shown in Figure 3. Compared with the two IR spectra, there were main peaks of PS, 1450 cm^−1^, 1490 cm^−1^, and 1600 cm^−1^ assigned to benzene ring and 2846 cm^−1^ and 2923 cm^−1^ to -CH_2_- groups. The peaks at 1733 cm^−1^ and 3415 cm^−1^ which only appeared in the CAPS spectrum were attributed to carboxyl groups, and they were not strong due to low amount of these groups being merely on the surface. Similarly, the bending vibration of other bonds in CAPS, C-N, broadened the peak width at 756 cm^−1^ assigned to the bending vibration of C-H (benzene ring).

### 3.2. Enrichment and Determination of Trace Metal Ions in “Standard” Seawater

**Figure 4:**
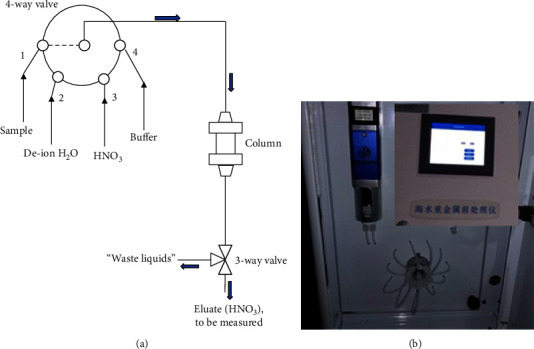
(a) Illustration of trace metal ion preconcentration system. (b) A digital photo of the laboratory that assembled the preconcentration unit.

To evaluate the novel resin filled in the column, 10 mL of standard seawater containing trace metals of Cu (5.0 *µ*g/L), Zn (70 *µ*g/L), Pb (10.0 *µ*g/L), Cd (1.0 *µ*g/L), and many more other kinds of ions, such as sodium, potassium, and so on, was flowed by 1 mL/min through the column, and 10 mL of the HNO_3_ eluant was collected for flame/graphite furnace AAS analysis, as shown in Figure 4. At the same time, the same amount of seawater was processed and complexed by APDC/DDTC based on the specification for marine monitoring–Part 4: Seawater analysis (GB 17378.4–2007).

The concentrated metal ion solution was measured on the AAS instrument, and the results are shown in Table 2. The modified PS beads and the preconcentrating device exhibit the same detection results as all that the APDC/DDTC process does.

**Figure 5:**
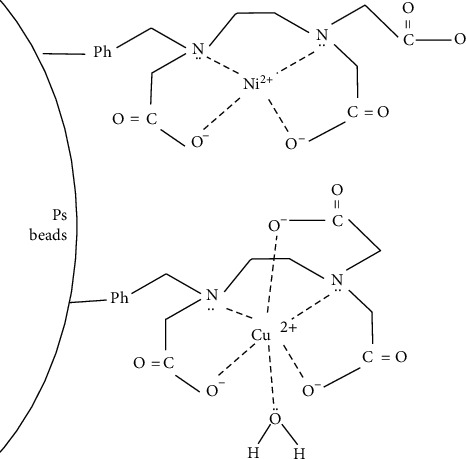
Trace metals ions chelated with amino polycarboxylic groups on the surface of modified PS beads.

When all the trace metals ions flowed through the column, trace metal ions were adsorbed and chelated with amino polycarboxylic groups on the surface of modified PS beads, as shown in Figure 5. These ions could be desorbed easily when HNO_3_ solution purged the same column and the HNO_3_ eluent containing the ions was sent to the AAS instrument for detection. The contents of the ions given by AAS were coincided with their standard values, which showed that the chelating resin of modified PS beads was effective on enriching the trace ions. After the HNO_3_ eluent ended, at the same time, the column was regenerated and could be used in the next enrichment process.

### 3.3. Investigation on Enrichment Efficiency

Generally, the seawater quality in the world is improving, and especially, the contents of heavy metals ion are decreasing, such as coastal waters of China. The standard seawater was diluted to 1/2, 1/4, 1/8, and 1/10 to evaluate the enrichment efficiency. The concentrations were shown in Table 3, in which these values were given by AAS and then multiplied by 2, 4, 8, and 10, respectively.

**Figure 6:**
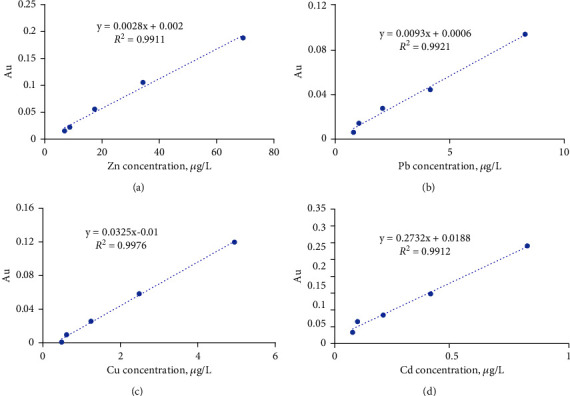
The relationship of absorbance and concentration of Zn (a), Pb (b), Cu (c), Cd (d), and fitting curve and correlation coefficient.

As shown in Figure 6, the correlation coefficient of this method for Zn, Pb, Cu, and Cd was 0.9911, 0.9921, 0.9976, and 0.9912, respectively, which stated clearly that the enriching process was efficient.

The LODs of Cu, Zn, Pb, and Cd were given, as shown in Table 4, which indicated that the developed enrichment method had high efficiency. Accordingly, the LODs of metal ions were lower than those given by APDC/DDTC treatment (GB 17378.4-2007).

**Figure 7:**
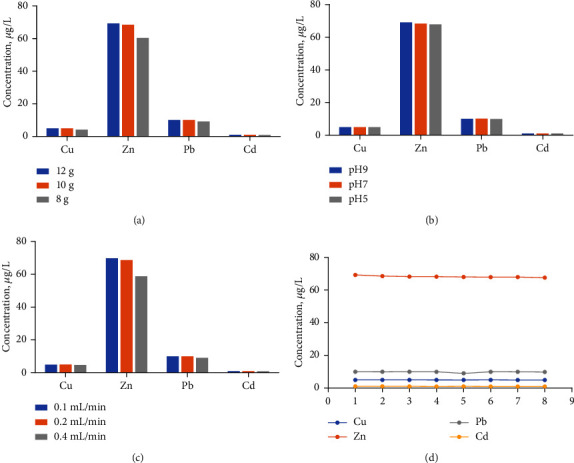
The effect of (a) mass of resin, (b) the flow rate of the sample, and (c) pH of the sample on the test results of trace metals. (d) Repeatability of the enrichment preconcentration column when applying the condition of 12 g resin, 0.2 mL/min of the flow rate, and pH 7.

To explore the effect of resin mass, the flow rate and pH of the water sample on the enriching ability or capacity of trace metals and the different enrichment conditions were adopted and testing results as shown in Figure 7. The mass of resin was more important for preconcentration, and at least 10 g was suitable for 20 mL of standard seawater. Compared with other kinds of resin, [4] the quantity of usage was much higher, which could be attributed to the lower specific surface area of commercial PS beads with big diameter and the grafted EDTA-type absorption groups on the surface. 0.2 mL/min was suitable for the enriching process (Figure 7(b)), and high speed, 0.4 mL/min easily caused the incomplete absorption of trace metals. The enriching capacity was not affected obviously by pH value of samples Figure 7(c). The enriching process was repeated 8 times to verify the resin's absorption ability, which showed good results (Figure 7(d)).

### 3.4. Applications in Detection of Waters

**Figure 8:**
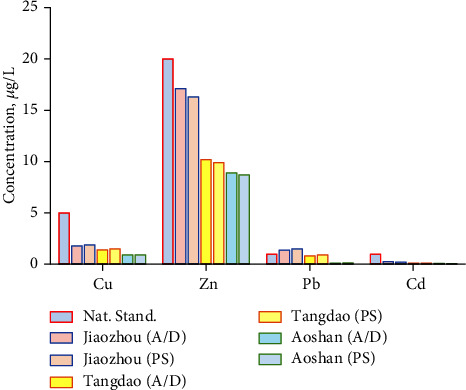
Actual measurement of seawater samples taken in three bays, Jiaozhou, Tangdao, and Aoshan, coastal sea of Qingdao, China, and conducting the enriching process for AAS to afford the concentration of Cu, Zn, Pb, and Cd.

The seawater samples were taken in Jiaozhou Bay, Tangdao Bay, and Aoshan Bay of Qingdao costal sea, Shandong, China. They were detected by the newly developed enriching method and, in contrast, also detected by means of APDC/DDTC treatment. As shown in Table 5 and Figure 8, the results were close, and all the seawater satisfied the standard of National class I sea water in China. Compared with the open sea of Tangdao Bay and Aoshan Bay, the continental sea, the water quality of Jiaozhou bay was the worst, and Pb content exceeded the National Standard in sampling time.

## 4. Conclusions

In general, a novel chelating resin, synthesized from commercial cross-linking PS beads modified by EDTA-type, amino polycarboxylic groups, was fabricated facilely and applied as a column filler to enrich trace metals, Cu, Zn, Pb, and Cd and to remove light metals, such as Na^+^ with high content, in seawaters, which eliminated interference when trace metals were analyzed on AAS (flame/graphite furnace). Correspondingly, a preconcentrating device was self-built based on this column and standard seawater introduced to evaluate this enriching filler and device. Some important parameters were investigated and optimzied, such as mass of resin, flow rate, and pH of the sample. More than 10 g of resin and 0.2 mL/min of flow rate were more suitable for preconcentrating trace metals from 20 mL of seawater, but pH value did not affect obviously the detecting results. Compared with the current organic extraction agents, APDC/DDTC, applied in GB 17378.4-2007, the modified PS resin beads exhibited higher enriching efficiency, lower LOD, and good repeatability, and the device had strong anti-interference ability and low cost, which could be directly applied for routine water detection.

## Figures and Tables

**Table 1:** Different enrichment conditions.

No.	Resin mass/g	Flow rate, mL/min	pH
1	8	0.2	7
2	10	0.2	7
3	12	0.2	7
4	12	0.1	7
5	12	0.2	7
6	12	0.4	7
7	12	0.2	7
8	12	0.2	8
9	12	0.2	9

**Table 2:** Detection of commercial standard seawater.

	Standard seawater, *µ*g/L	Enriched by APDC/DDTC, *µ*g/L	Enriched by modified PS beads, *µ*g/L
Cu	5.0 ± 0.4	5.103	4.949
Zn	70 ± 3	71.211	69.248
Pb	10.0 ± 0.6	9.974	9.951
Cd	1.00 ± 0.06	0.971	0.987

**Table 3:** Diluted standard seawater.

Metals	Standard seawater, *µ*g/L	1/2, *µ*g/L		1/4, *µ*g/L		1/8, *µ*g/L		1/10, *µ*g/L	
		A/*D*^1^	PS^2^	A/*D*	PS	A/*D*	PS	A/*D*	PS
Cu	5.0 ± 0.4	2.490	2.489	1.248	1.245	0.620	0.615	0.511	0.485
Zn	70 ± 3.0	35.01	34.31	17.48	17.43	8.762	8.729	7.053	6.901
Pb	10.0 ± 0.6	4.982	4.987	2.492	2.486	1.251	1.243	0.981	0.962
Cd	1.00 ± 0.06	0.495	0.491	0.251	0.248	0.120	0.116	0.097	0.091

^1^A/*D*: APDC/DDTC treatment; ^2^PS: CAPS preconcentration.

**Table 4:** LOD of Cu, Zn, Pb, and Cd.

Metals	LOD by APDC/DDTC, *µ*g/L	LOD by modified PS beads, *µ*g/L
Cu	0.2	0.2
Zn	4.0	3.1
Pb	0.08	0.03
Cd	0.02	0.01

**Table 5:** Detection of trace metals of seawaters (Samples taken in Dec. 2021).

Ions	National class I sea water/*µ*g/L	Jiaozhou Bay (Hongdao)/*µ*g/*L*		Tangdao Bay (Huangdao)/*µ*g/L		Aoshan Bay (Jimo)/*µ*g/L	
		A/*D*	PS	A/*D*	PS	A/*D*	PS
Cu	5	1.806	1.891	1.411	1.505	0.920	0.889
Zn	20	17.074	16.341	10.212	9.923	8.897	8.718
Pb	1	1.380	1.495	0.833	0.904	0.121	0.129
Cd	1	0.252	0.213	0.124	0.103	0.084	0.073

## Data Availability

The data that support the findings of this study are available from the author upon reasonable request.
